# Implementation of a prospective neurosurgical complication registry in routine clinical practice

**DOI:** 10.1007/s00701-026-06965-6

**Published:** 2026-07-10

**Authors:** Anja Grimmer, Kristina-Maria Geley-Eder, Petra Böhm, Vanessa Seiter, Baran Atli, Anna Hauser, Kathrin Aufschnaiter-Hiessböck, Tobias Rossmann, Eva Horner, Matthias Gmeiner, Michael Giretzlehner, Andreas Gruber, Maria Gollwitzer

**Affiliations:** 1 Department of Neurosurgery, Kepler University Hospital, Johannes Kepler University Linz, Linz, Austria; 2 Johannes Kepler University Linz, Linz, Austria; 3 RISC Software GmbH, Hagenberg, Austria; 4 Clinical Research Institute for Neuroscience, Johannes Kepler University Linz, Linz, Austria

**Keywords:** Quality improvement, Clinical registry, Process evaluation, Workflow integration, Health information systems

## Abstract

**Background:**

Reliable and complete documentation is a prerequisite for quality improvement in neurosurgical care. To strengthen internal process monitoring and reduce variability in documentation practices, our department implemented a structured, web-based neurosurgical complication documentation and quality assurance registry combining automated data imports from the hospital information system with minimal standardized manual inputs. The year 2024 represented the first full operational cycle of the system.

**Methods:**

This quality-improvement study evaluated exclusively process-related parameters of the complication documentation registry and did not include analysis of clinical content or outcome variables. Although the registry contains prospectively documented clinical and treatment-related data, the present evaluation was limited to system integration, documentation completeness, frequency of missing mandatory documentation, and performance of reporting mechanisms. Analyses were based solely on de-identified system-level metadata reflecting documentation status. Documentation completeness and workflow adherence were analyzed descriptively over time.

**Results:**

The registry was successfully integrated into routine clinical workflow across all neurosurgical units and across all hospital wards responsible for the management or discharge of neurosurgical patients. Documentation completeness improved steadily throughout the year, increasing from 77% in the first quarter to 88% in the fourth quarter when assessed after a predefined follow-up interval. Automated reporting identified incomplete documentation entries primarily during the early implementation phase, with alert frequencies decreasing by approximately 48% over the course of the year. Documentation completeness continued to improve beyond the formal observation period, and all inpatient stays from the 2024 cohort were fully documented during follow-up. Progressive improvements across units indicated growing feasibility, acceptance, and compliance within the clinical teams.

**Conclusion:**

The structured multi-step design, combining automated data integration, binary discharge screening, and dedicated detailed documentation by designated clinicians, appeared effective in improving documentation completeness and workflow adherence. This process-oriented evaluation provides a useful structural basis for future quality improvement initiatives and ethically approved clinical research based on high-fidelity routinely collected data.

## Introduction

High-quality and standardized documentation is a fundamental requirement of modern neurosurgical practice and a central component of continuous quality improvement. Foundational concepts of healthcare quality, including the systematic assessment of structure, process, and outcome measures, emphasize the importance of reliable data for monitoring performance and guiding organizational development [6]. In neurosurgery, where clinical pathways are complex and inherently multidisciplinary, complete and accurate complication documentation is essential for ensuring patient safety, identifying deviations from expected processes, and supporting institutional learning. Complication-related events, including unplanned readmissions, have been shown to represent meaningful quality indicators and targets for improvement initiatives in neurosurgical care [2].

Beyond technical performance, surgical quality depends on robust systems that allow consistent data capture and evaluation, particularly in routine clinical practice [9]. Despite its recognized importance, the implementation of structured and prospectively maintained documentation systems remains challenging. In surgical departments in particular, complication reporting is often perceived as sensitive, and implementation of additional documentation requirements may initially encounter limited acceptance. Heterogeneous workflows and inconsistent documentation practices can substantially reduce the utility of routinely collected institutional data. Evidence from neurosurgical registries demonstrates the value of longitudinal, standardized data collection for understanding practice patterns and enhancing transparency at the departmental level [5, 17]. However, the long-term success of such registries depends not only on technical solutions but also on sustainable processes and staff compliance [8].

Standardized complication classification systems, most prominently the Clavien–Dindo classification in general surgery [4], have substantially improved consistency in reporting surgical adverse events. In neurosurgery, the Landriel Ibañez framework represents a specialty-adapted grading system building upon similar principles [12]. However, their effectiveness depends on reliable prospective documentation in routine clinical practice. Structured workflow optimization and feedback mechanisms may facilitate integration of documentation tasks into daily neurosurgical practice [7, 13, 14].

More broadly, methodological standards for studies using routinely collected health information emphasize data completeness, transparency in data handling, and clear description of data flows as prerequisites for robust internal quality assessment [1, 18]. These principles apply equally to institutional quality improvement initiatives, where the validity of internal evaluations is directly dependent on the reliability of underlying documentation processes. Surgical quality increasingly relies on structured systems enabling continuous monitoring of routine clinical practice [20].

Against this background, our department implemented a structured, web-based neurosurgical complication documentation and quality assurance registry designed to combine automated data imports from the hospital information system with minimal, standardized manual inputs. The registry was developed to prospectively capture neurosurgical complications within a structured framework integrating established grading concepts and institution-specific categories. The year 2024 represents the first full cycle of routine operation, providing an opportunity to evaluate system implementation, completeness of mandatory documentation fields, and integration into daily clinical workflows.

In contrast to clinical outcome studies, the present work focuses exclusively on process-related aspects of the registry infrastructure. By evaluating its structure, documentation completeness, and workflow adherence, this study aims to assess the feasibility of implementing structured complication documentation in neurosurgical departments.

## Methods

This study was designed as an institutional quality improvement and process evaluation project and did not involve analysis of patient-level clinical outcomes or detailed clinical variables beyond those required for structured documentation workflows. The methodological focus was limited to assessing the implementation, structural performance, workflow integration, and documentation completeness of a newly introduced neurosurgical complication documentation and quality assurance registry during its first full operational year (January–December 2024), which also represented the initial user adaptation phase. Because the analysis was limited to de-identified system-level metadata reflecting documentation status and did not include evaluation of patient-identifiable or outcome-related clinical variables, formal ethics approval was not required.

The registry was designed to prospectively document neurosurgical complications and related quality assurance parameters for all inpatient encounters within the department. Its data structure integrates elements of established neurosurgical complication grading systems, including the Landriel Ibañez classification [12], while incorporating institution-specific categories such as intraoperative events, postoperative complications, infectious complications, and relevant medical adverse events. The present study did not analyze these clinical variables but focused exclusively on documentation processes and system performance.

Complications were defined as any undesirable clinical event occurring during hospitalization that required additional diagnostic or therapeutic measures or resulted in neurological deterioration. This definition also included complication-related readmissions linked to previous neurosurgical interventions.

Within the registry, complications are prospectively documented using a structured categorization framework. For each documented complication, the corresponding severity grade is automatically calculated in the background based on the presence of neurological deterioration and the type of treatment required for complication management.

Severity grading follows the Landriel Ibañez classification system for neurosurgical complications, conceptually aligned with the Clavien–Dindo classification in general surgery and thereby allowing comparability across surgical disciplines and institutions. For each patient encounter, a final complication grade ranging from Grade I to Grade IV is determined. In cases where multiple complications occur during a single hospital stay, the highest severity grade is used to represent the overall complication classification for that encounter.

The classification algorithm integrates predefined clinical parameters, including the presence of multi-organ failure, need for intensive care treatment, and the type of therapeutic intervention required to manage the complication (e.g., pharmacological treatment, invasive intervention with or without anesthesia).

In addition to severity grading, complications are categorized into predefined groups to ensure standardized documentation and reproducibility. These categories include intraoperative or interventional complications, postoperative complications, surgical site infections, relevant medical complications, complications in conservatively treated patients, and death. Each category is further defined by specific diagnostic and clinical criteria, including radiological findings, microbiological confirmation, need for therapeutic intervention, and occurrence of revision surgery. This structured approach allows detailed and consistent characterization of complications while maintaining feasibility in routine clinical practice.

A detailed internal classification framework with predefined subcategories and diagnostic criteria was used to standardize data entry, minimize variability between users, and ensure reproducibility of documentation.

The documentation system is based on a structured interaction between the hospital information system (KIS), a data management platform (Calumma; RISC Software GmbH, Hagenberg, Austria), and a dedicated reporting tool. At patient discharge, the responsible clinician is required to answer two predefined mandatory screening questions within the KIS, indicating whether a complication occurred during the hospital stay and whether the current admission was related to a complication from a previous encounter. These binary screening questions determine whether additional quality assurance documentation is required. The simplified yes/no structure was deliberately chosen to minimize documentation burden and to reduce the risk of underreporting related to documentation workload. This two-step approach operationalizes complication reporting by ensuring mandatory screening of all inpatient encounters at discharge, followed by structured documentation of complications in positively screened cases.

To minimize manual workload, all discharge records are transferred into the Calumma platform on a daily basis. Automated imports include admission and discharge records, intra-hospital transfers, metadata on operative or interventional procedures, and the responses to the two screening questions. Imported data serve exclusively as structural placeholders and were not analyzed as clinical variables.

The Calumma platform provides a web-based interface accessible on all neurosurgical wards and associated intensive care units within the NeuroMed Campus. The platform is hierarchically structured, with patient, hospital stay, and treatment episodes forming the core elements of the data model. For the present analysis, however, no patient-level variables were accessed; only the presence or absence of mandatory administrative documentation fields and the internal status of each dataset were evaluated.

Manual documentation is performed in two predefined steps. In the first step, completed at discharge within the KIS, the two mandatory screening questions are answered for all inpatient encounters. In the second step, which is only triggered if at least one screening question is answered affirmatively, designated senior residents or attending physicians complete supplementary documentation within the Calumma platform. Assignment of treatment modality (conservative, operative, or interventional) is performed automatically based on imported metadata and does not require manual input. Dataset completeness is subsequently verified using a dedicated interface identifying missing documentation. The two-step structure represents the central quality mechanism of the registry. Step 1 requires minimal mandatory input at discharge and ensures complete case screening with negligible additional workload. Step 2, performed by designated and trained clinicians, allows structured and detailed documentation while maintaining accountability and data consistency. All complication entries were subsequently reviewed by designated neurosurgeons to improve completeness and internal consistency of documentation and to correct obvious documentation errors when necessary.

To support workflow adherence and continuous quality monitoring, a unified reminder system is employed. Weekly reminders identify encounters with unanswered screening questions and encounters requiring completion of supplementary documentation. In addition, monthly aggregated summary reports are generated, providing an overview of all encounters with missing mandatory administrative documentation fields as well as descriptive analyses of completed documentation entries. These reporting mechanisms support both operational follow-up and higher-level assessment of documentation performance. In addition, predefined query-based monitoring tools were implemented to systematically identify incomplete documentation, including missing screening information, incomplete complication entries, and missing therapy data. This enabled targeted follow-up and continuous validation of documentation completeness across clinical units. Together, these measures were used as process indicators to assess the effectiveness of reporting over time.

No detailed patient-level clinical characteristics or outcome variables were analyzed. All data used were de-identified registry-derived variables related to documentation status, screening responses, and workflow completion metrics. No clinical content variables were analyzed. Analyses were performed using simple descriptive metrics generated by a dedicated reporting tool based on structured metadata stored within the Calumma platform. No statistical inference or comparative analysis was conducted. Correctness of individual complication classifications as well as potential under- or overreporting were not systematically evaluated and remain subjects for future validation studies.

Outcome measures were limited to process-related indicators derived from the documentation workflow. Completion of step 1, defined as the completion of the two mandatory screening questions, was evaluated across all imported inpatient discharges. Completion of step 2, defined as supplementary documentation within the Calumma platform, was assessed only for the subset of encounters in which at least one screening question was answered affirmatively in step 1.

## Results

During the first full year of operation (January–December 2024), the neurosurgical documentation system was applied to all inpatient discharges across neurosurgical wards and associated intensive care units. A total of 3,945 discharged inpatient stays were registered in the platform through automated imports from the hospital information system. For all discharges, completion of the two mandatory screening questions was mandatory at discharge.

Among these discharged stays, 462 (11.7%) had at least one screening question answered affirmatively, thereby triggering the requirement for supplementary documentation in a second documentation step.

**Fig. 1:**
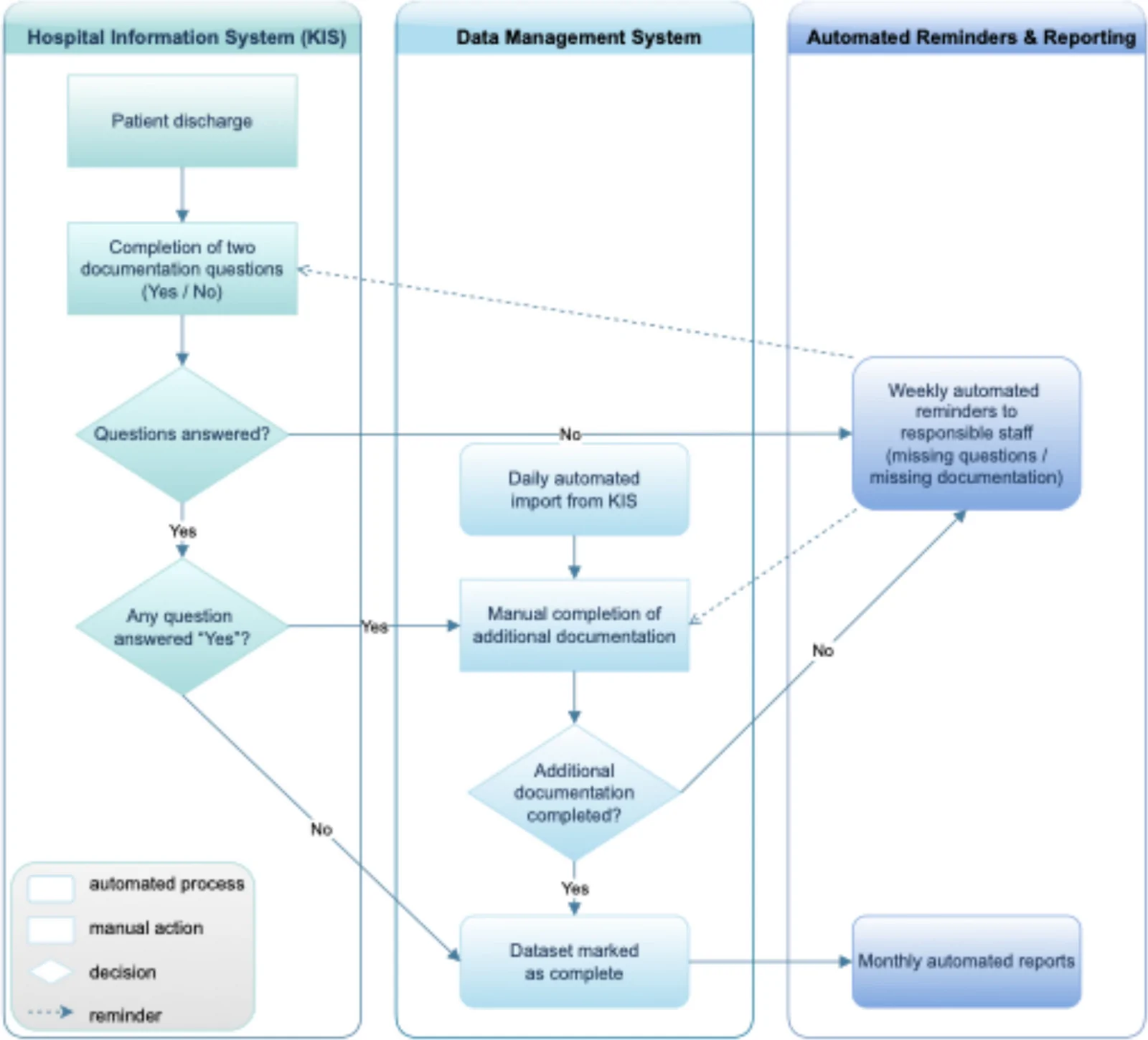
Workflow of the neurosurgical documentation system. Figure illustrates the structured workflow of the neurosurgical quality assurance and documentation system. At discharge, two mandatory screening questions are completed within the hospital information system (KIS) for all inpatient stays, indicating whether a complication occurred during the hospital stay and whether the admission was related to a complication from a previous encounter (Step 1). Discharge data, including responses to the screening questions, are transferred automatically to the documentation platform (Calumma). Encounters with at least one affirmative screening response trigger a second documentation step (Step 2), requiring supplementary administrative documentation. Automated weekly reminders and monthly aggregated reports identify incomplete screening responses and missing supplementary documentation, supporting continuous process monitoring and workflow adherence

The structured documentation workflow and the dependency between the two documentation steps are illustrated in Fig. 1.

### System adoption and use across clinical units

The documentation system was integrated into routine workflow across all neurosurgical units and across all hospital wards responsible for the management or discharge of neurosurgical patients. Access logs demonstrated consistent usage throughout the year, with a gradual increase in the number of finalized datasets per month. Based on quarterly evaluations performed with a predefined follow-up interval after discharge, the proportion of encounters with complete documentation increased from 77% in the first quarter to 88% in the fourth quarter of 2024.

Documentation completeness continued to improve beyond the formal observation period. By July 2025, all inpatient stays from the 2024 cohort had been fully documented, resulting in complete documentation coverage within the registry workflow.

**Fig. 2:**
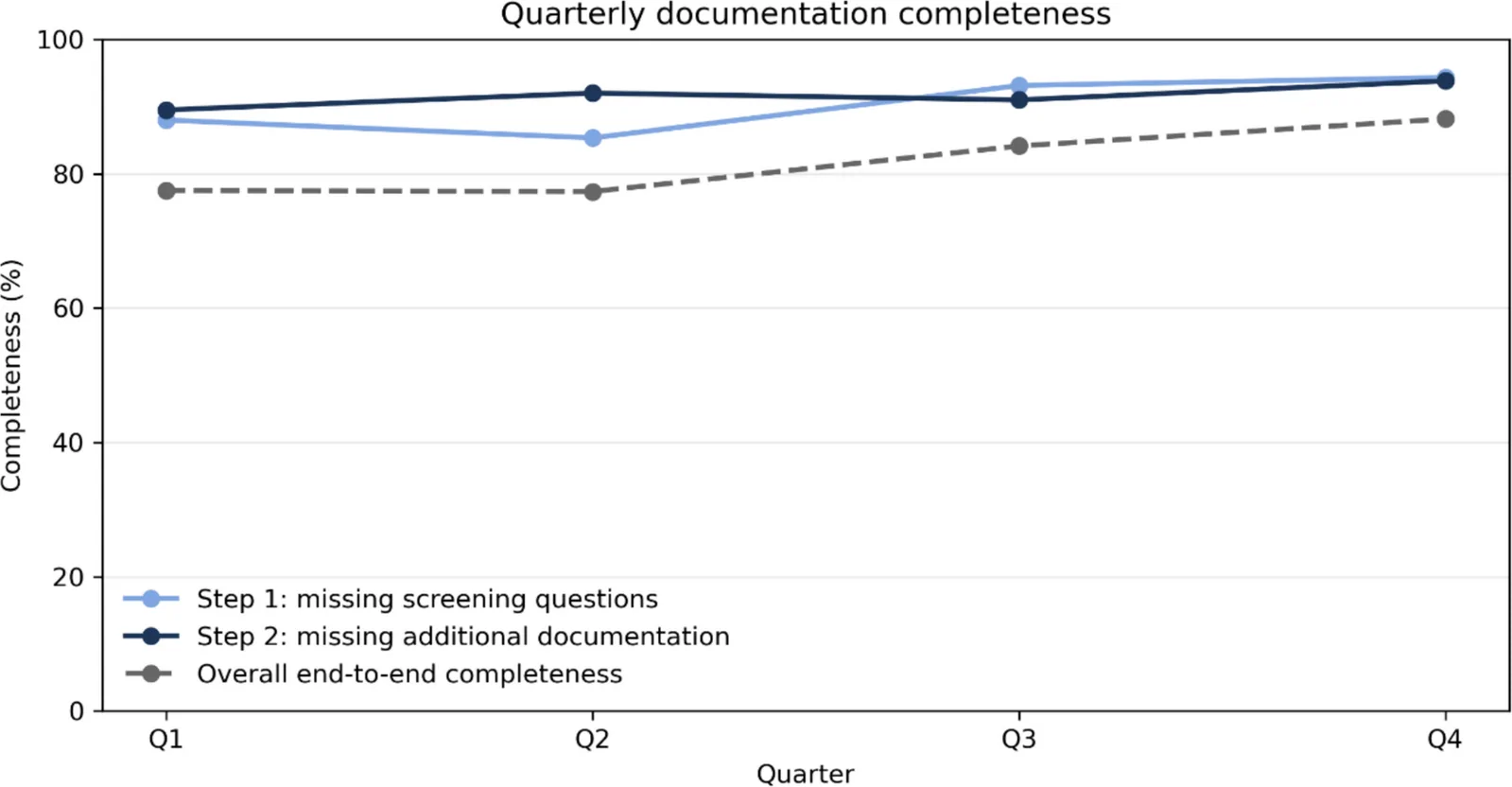
Quarterly trend in documentation completeness. Figure shows the proportion of inpatient discharges with completed mandatory documentation fields over the course of 2024, assessed on a quarterly basis. Completeness was evaluated after a predefined follow-up interval following discharge to account for delayed completion of documentation. A continuous increase in documentation completeness was observed throughout the year, with further completion of remaining datasets occurring after the formal observation period

The quarterly trend in documentation completeness is illustrated in Fig. 2.

### Completeness of mandatory fields

**Fig. 3:**
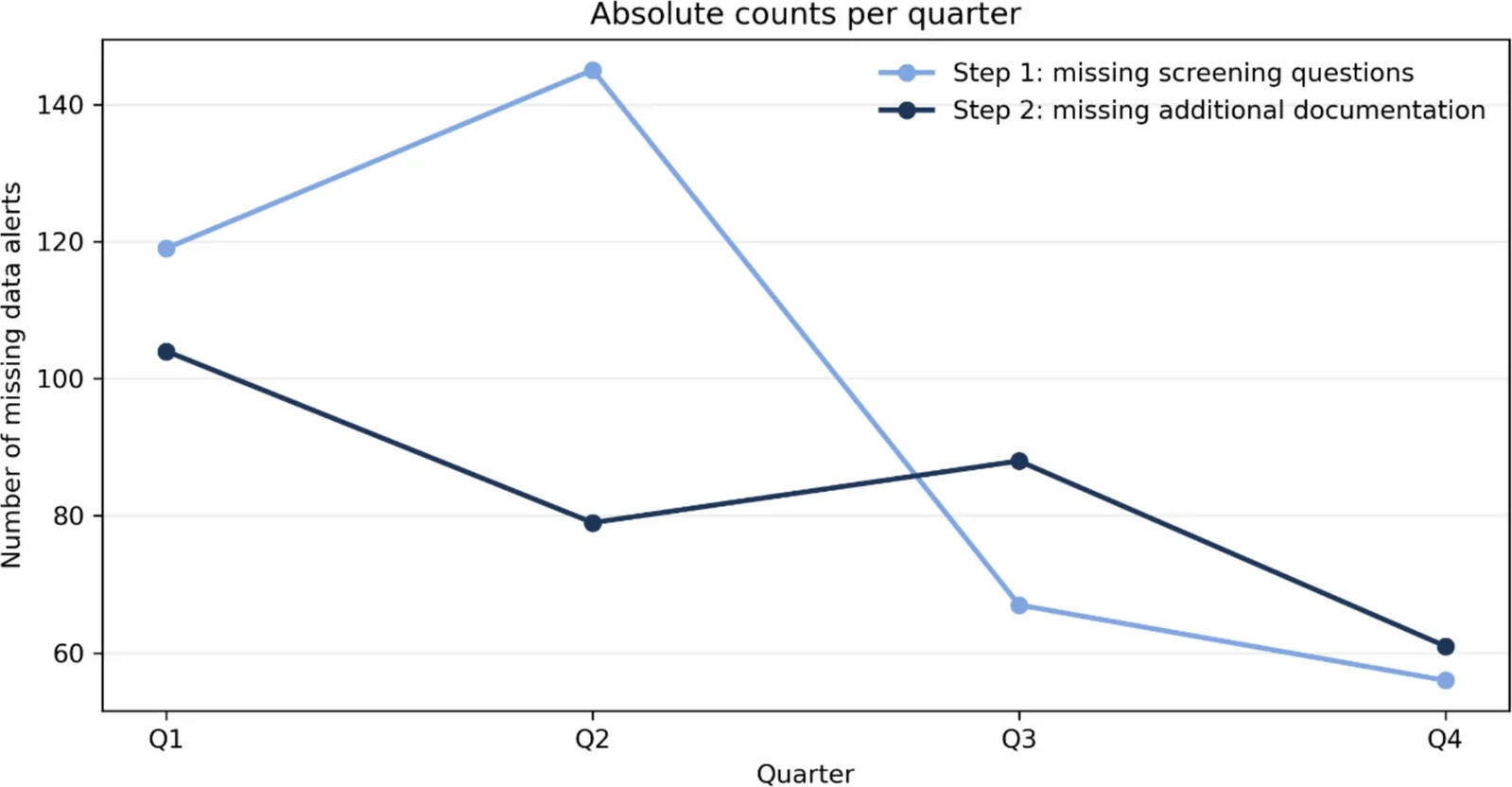
Quarterly trend of incomplete documentation. Figure depicts the quarterly distribution of incomplete documentation entries identified through automated reports during 2024. Incomplete documentation includes missing completion of step 1 screening questions at discharge as well as missing step 2 supplementary documentation for encounters with at least one affirmative screening response. A peak in incomplete documentation was observed during the second quarter, followed by a continuous decline over subsequent quarters, consistent with increasing workflow adherence and system familiarization

At the beginning of the observation period, 12% of discharged inpatient stays lacked completion of step 1 documentation, defined as the two mandatory screening questions completed at discharge. Over the course of the year, this proportion decreased to 6%, corresponding to a relative reduction of 50%. In total, 387 instances of incomplete step 1 documentation were identified through automated reports, with the highest number occurring in May (*n* = 78), followed by a steady decline over subsequent months. Quarterly trends in incomplete documentation identified through automated reports are shown in Fig. 3.

Among inpatient stays requiring step 2 documentation, defined as supplementary documentation triggered by at least one affirmative screening response in step 1, 83% were incomplete at baseline. This proportion decreased to 63% by the end of the observation period. Documentation completeness continued to improve beyond the formal study period, and by mid-2025 all inpatient stays from the 2024 cohort had been fully documented, resulting in 100% completeness.

### Performance of reporting tools

Automated reporting appeared to contribute substantially to improving documentation completeness. During the 12-month observation period, a total of 332 alerts indicating incomplete supplementary documentation were generated, while alerts related to missing mandatory screening documentation corresponded to those described above.

Across both alert categories, the number of alerts decreased substantially after the second quarter, coinciding with increasing staff familiarization and routine integration of the documentation workflow. By the final quarter of 2024, alert counts had decreased by 48% compared to the initial observation period.

### Ward-level variation

Documentation completeness varied between clinical units but improved consistently across all wards and discharge locations. When considering all neurosurgical wards, intensive care units, and additional discharge stations, completeness rates in the first quarter ranged from 6 to 87%. By the fourth quarter, this range had narrowed substantially, reaching 31% to 96%.

**Fig. 4:**
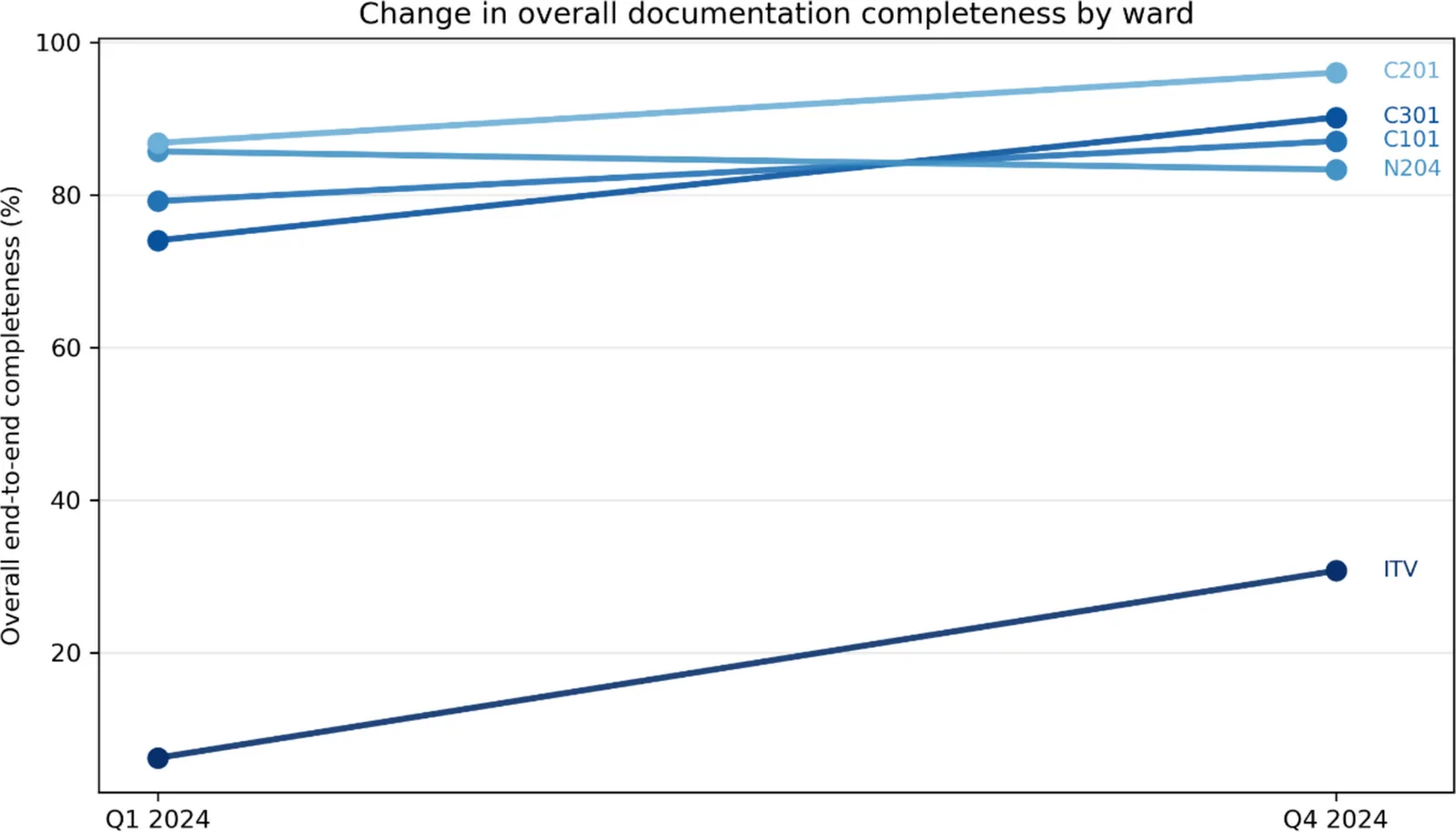
Ward-level variation in documentation completeness. Figure illustrates documentation completeness across individual neurosurgical wards, intensive care units, and additional discharge stations over the course of 2024. Completeness is shown as the proportion of inpatient discharges with completed mandatory documentation fields, assessed quarterly. While substantial variation between units was observed during the initial observation period, documentation completeness improved across all units over time, with a marked narrowing of the observed range by the fourth quarter

The most pronounced improvements were observed in high-throughput neurosurgical wards, where the number of incomplete documentation alerts decreased markedly over the observation period, consistent with increasing workflow adherence across units (Fig. 4).

### Workflow integration

Qualitative feedback obtained during routine internal meetings suggested increasing user familiarity with the documentation system over time. Informal qualitative feedback from clinicians suggested that, following an initial adaptation phase, the system did not result in a perceived increase in administrative workload. The separation of responsibilities, with ward physicians completing the initial screening documentation and senior clinicians responsible for supplementary documentation and verification, was frequently perceived as supportive of workflow integration. Automated weekly reminders and monthly aggregated summary reports were considered useful tools for maintaining documentation completeness without the need for continuous manual oversight.

## Discussion

The implementation of a structured neurosurgical complication documentation and quality assurance registry in our department demonstrated that high levels of documentation completeness, workflow integration, and institutional acceptance are achievable within a relatively short period. In line with Donabedian’s foundational framework, which emphasizes the interdependence of structure, process, and outcome in healthcare quality, our findings suggest that robust documentation processes represent an important structural prerequisite for reliable assessment of outcomes and complications in neurosurgical practice [6]. By deliberately focusing on process-related indicators rather than clinical variables, this study provides insight into the feasibility and sustainability of integrating a documentation system into routine neurosurgical practice.

A key observation was the continuous improvement in documentation completeness throughout the observation period. This finding is consistent with evidence from quality-improvement research showing that structured workflows and clearly defined responsibilities enhance data quality and reduce variability in documentation practices [13, 14]. In our setting, the use of automated reporting tools to identify incomplete documentation proved particularly effective. Such feedback mechanisms reflect core principles of the RECORD statement, which emphasizes transparency, traceability of data flows, and internal consistency when working with routinely collected health information [1]. Within a quality-management framework, these reports function as real-time process control tools rather than retrospective audits [21]. The transparent attribution of documentation status within the system supports individual accountability and contributes to sustained compliance. The effectiveness of the documentation workflow was evaluated through several predefined process indicators, including completion rates of mandatory documentation fields, frequency of automated missing-data alerts, and temporal trends in documentation completeness across clinical units.

The progressive reduction in missing documentation alerts across clinical units suggests successful integration of the system into daily workflow. Previous work has highlighted a persistent implementation gap in surgery, whereby evidence and quality initiatives often fail to translate into routine clinical practice without adequate local infrastructure and monitoring mechanisms [19]. In the absence of a universally established neurosurgical complication classification system, the Landriel Ibañez framework has been widely adopted and adapted for structured severity grading^2^. Similarly, our results indicate that predefined structures and standardized decision pathways can enhance consistency and completeness of routine documentation processes. Although clinical complications were intentionally not analyzed in this study, the results suggest that important structural prerequisites for high-quality data capture are in place. Established complication grading systems such as the Clavien-Dindo classification and its neurosurgical adaptation by Landriel Ibañez have demonstrated that standardized documentation is essential for meaningful comparison and analysis of surgical outcomes across institutions. A key design feature contributing to data quality was the two-step documentation model. By separating universal screening from detailed documentation and assigning responsibilities to specifically designated clinicians, the system minimized entry barriers while preserving structured data integrity. The possibility of completing step 2 documentation independently of physical ward presence further supported sustainability of the workflow.

Experience from neurosurgical registries further supports the notion that long-term data quality depends on both technical infrastructure and sustained user engagement [15, 17]. The hybrid design of our system, combining automated data imports with minimal manual input, appears to achieve a pragmatic balance between usability and completeness. Embedding documentation tasks into existing discharge workflows likely contributed to increasing user acceptance over time.

Importantly, our findings suggest that effective documentation infrastructure can be established without imposing a substantial administrative burden on clinical staff. Prior work on process mapping in neurosurgery has highlighted workflow clarity and user ownership as key determinants of successful quality-improvement initiatives [13]. Consistent with these observations, the separation of responsibilities between ward-based screening documentation and subsequent verification by senior clinicians may have been a critical factor supporting sustainable workflow integration in our department.

Several implications arise from this evaluation. Evaluations of local surgery-related quality improvement initiatives have shown that sustainable improvements are closely linked to structured processes and continuous monitoring rather than isolated outcome assessment [11]. First, early investment in structural integration, particularly automated interfaces with hospital information systems, appears to reduce long-term workload while improving data fidelity [16]. Second, routine reporting and transparent feedback loops are essential for maintaining documentation quality over time [3]. Third, focusing initially on documentation processes rather than clinical outcomes provides a stable foundation for future quality-improvement initiatives and ethically approved research [10]. Once documentation structures are reliable, subsequent analyses of clinical variables can be conducted with greater confidence in the underlying data integrity.

This study has several limitations. The analysis was restricted to process-related indicators and did not include clinical or patient-level data, limiting conclusions regarding the direct impact of documentation completeness on clinical outcomes. Furthermore, reliance on system-generated metadata means that improved completeness does not necessarily equate to improved accuracy or granularity of clinical content. In addition, correctness of individual complication classifications as well as potential under- or overreporting were not systematically assessed. Although complication entries underwent secondary review by designated neurosurgeons, formal validation of interobserver reliability and classification accuracy remains an important objective for future studies. As a single-center experience, our findings may reflect local organizational factors and may not be fully generalizable to other neurosurgical settings. The establishment of a broadly accepted specialty-specific documentation standard could further enhance comparability between institutions and support multicenter quality improvement efforts.

In summary, this study demonstrates that a structured neurosurgical documentation system can be feasibly integrated into routine clinical workflow, achieving progressive improvements in documentation completeness over its first operational year. By emphasizing structure and process, the system provides a useful foundation for sustainable quality management and future research initiatives.

## Conclusion

The implementation of a structured, web-based neurosurgical documentation system appeared feasible and sustainable within routine clinical practice. During the first full operational year, the system achieved progressive improvements in documentation completeness, supported by automated data imports, minimal manual input requirements, and structured reporting mechanisms. These findings suggest that structured prospective documentation can be integrated into daily neurosurgical workflow without substantial perceived additional administrative burden. The structured multi-step design, consisting of minimal routine screening at discharge followed by detailed documentation performed by designated clinicians, appears to support acceptance while improving data consistency and completeness within the registry workflow.

By focusing on structural and process-related indicators rather than clinical outcomes, this evaluation provides a useful structural foundation for future quality improvement initiatives and ethically approved clinical research based on high-fidelity routinely collected data. The experience presented here may provide a practical framework for other neurosurgical centers aiming to strengthen documentation infrastructure and enhance the reliability of institutional data.

## Data Availability

The data used in this study are not publicly available due to institutional data protection regulations, as they were generated within routine clinical quality management processes.

## References

[1] Benchimol EI, Smeeth L, Guttmann A, Harron K, Moher D, Petersen I, Sørensen HT, von Elm E, Langan SM (2015) RECORD working committee. The REporting of studies Conducted using Observational Routinely-collected health Data (RECORD) statement. PLoS Med. 12(10):e1001885. 10.1371/journal.pmed.1001885 PMC4595218 26440803

[2] Buchanan CC, Hernandez EA, Anderson JM et al (2014) Analysis of 30-day readmissions among neurosurgical patients: surgical complication avoidance as key to quality improvement: Clinical article. J Neurosurg 121(1):170–175. 10.3171/2014.4.JNS13944 24834942

[3] Byrne BE, Faiz OD, Bottle A et al (2021) A protocol is not enough: enhanced recovery program-based care and clinician adherence associated with shorter stay after colorectal surgery. World J Surg 45(2):347–355. 10.1007/s00268-020-05810-w PMC7773614 33079245

[4] Clavien PA, Barkun J, de Oliveira ML, Vauthey JN, Dindo D, Schulick RD, de Santibañes E, Pekolj J, Slankamenac K, Bassi C, Graf R, Vonlanthen R, Padbury R, Cameron JL, Makuuchi M (2009) The Clavien-Dindo classification of surgical complications: five-year experience. Ann Surg 250(2):187–196. 10.1097/SLA.0b013e3181b13ca2 19638912

[5] Dao Trong P, Olivares A, El Damaty A, Unterberg A (2023) Adverse events in neurosurgery: a comprehensive single-center analysis of a prospectively compiled database. Acta Neurochir (Wien) 165(3):585–593. 10.1007/s00701-022-05462-w PMC10006024 36624233

[6] Donabedian A (1988) The quality of care. How can it be assessed? JAMA 260(12):1743–8. 10.1001/jama.260.12.1743 3045356

[7] Ferreira MY, Cardoso LJC, Günkan A, Hong A, Santos AB, Reis R, Gonçalves OR, Barros EA, Borges PGLB, Semione G, Paleare LF, Bocanegra-Becerra JE, Bertani R, Okon II, Ferreira C, Serulle Y (2024) Challenges and limitations in meta-analyses of complications in neurosurgery: Systematic review with proposed approach and checklist to mitigate errors and improve the assessment of the real-world experience. Neurosurg Rev 47(1):722. 10.1007/s10143-024-02952-6 39358561

[8] Giesbrecht V, Au S (2016) Morbidity and mortality conferences: a narrative review of strategies to prioritize quality improvement. Jt Comm J Qual Patient Saf 42(11):516–527. 10.1016/S1553-7250(16)42094-5 28266920

[9] Horton R (1996) Surgical research or comic opera: questions, but few answers. Lancet 347(9007):984–985. 10.1016/s0140-6736(96)90137-3 8606606

[10] Katsura M, Kuriyama A, Tada M et al (2021) High variability in results and methodological quality among overlapping systematic reviews on the same topics in surgery: a meta-epidemiological study. Br J Surg 108(12):1521–1529 10.1093/bjs/znab328 34791075

[11] Ko CY, Shah T, Nathens A et al (2022) How well is surgical improvement being conducted? Evaluation of 50 local surgery-related improvement efforts. J Am Coll Surg 235(4):573–580. 10.1097/xcs.0000000000000341 36102577

[12] Landriel Ibañez FA, Hem S, Ajler P, Vecchi E, Ciraolo C, Baccanelli M, Tramontano R, Knezevich F, Carrizo A (2011) A new classification of complications in neurosurgery. World Neurosurg 75(5–6):709–715. 10.1016/j.wneu.2010.11.010 21704941

[13] McLaughlin N, Upadhyaya P, Buxey F, Martin NA (2014) Value-based neurosurgery: measuring and reducing the cost of microvascular decompression surgery. J Neurosurg 121(3):700–708. 10.3171/2014.5.JNS131996 25014441

[14] McLaughlin N, Rodstein J, Burke MA, Martin NA (2014) Demystifying process mapping: a key step in neurosurgical quality improvement initiatives. Neurosurgery 75(2):99–109. 10.1227/NEU.0000000000000360 24681644

[15] Meyer HS, Wagner A, Obermueller T et al (2022) Assessment of the incidence and nature of adverse events and their association with human error in neurosurgery A prospective observation. Brain Spine 2:100853. 10.1016/j.bas.2021.100853 PMC9560675 36248119

[16] Pallari E, Khadjesari Z, Aceituno D et al (2021) Implementation and evaluation of quality improvement training in surgery: a systematic review. Ann Surg 274(6):e489-506. 10.1097/sla.0000000000004751 34784666

[17] Sarnthein J, Staartjes VE, Regli L; Neurosurgery-Registry consortium (2022) Neurosurgery outcomes and complications in a monocentric 7-year patient registry. Brain Spine 2:100860. 10.1016/j.bas.2022.100860. PMC9560692 36248111

[18] Schipmann S, Varghese J, Brix T et al (2019) Establishing risk-adjusted quality indicators in surgery using administrative data-an example from neurosurgery. Acta Neurochir (Wien) 161(6):1057–1065. 10.1007/s00701-018-03792-2 31025177

[19] Schmidtke KA, Evison F, Grove A et al (2022) Surgical implementation gap: an interrupted time series analysis with interviews examining the impact of surgical trials on surgical practice in England. BMJ Qual Saf. 10.1136/bmjqs-2022-015077 PMC10314058 36270800

[20] Søreide K (2023) A formula for survival in surgery. Patient Saf Surg 17:13. 10.1186/s13037-023-00362-z PMC10225082 37245020

[21] Steiger HJ, Stummer W, Hänggi D (2010) Can systematic analysis of morbidity and mortality reduce complication rates in neurosurgery? Acta Neurochir (Wien) 152(12):2013–2019. 10.1007/s00701-010-0822-3 20936313

